# Infective Arthritis.—II

**Published:** 1911-03-25

**Authors:** W. D'Oyly Grange

**Affiliations:** President of the Harrogate Medical Society.


### Hospital Clinics.

### INFECTIVE ARTHRITIS.—II.

By W. D'OYLY GRANGE, M.D. Edin., President of the Harrogate Medical Society.

(Lecture delivered at the Polyclinic.)

In the pre-antiseptic days one saw a great deal of the pyæmic infection of joints, sometimes in a mild form, sometimes the virulent disease in all its awfulness. That is a form of infection of the joints which no one will deny. Then comes the gonorrhœal arthritis, called gonorrhœal rheumatism because we cannot get rid of the word rheumatism all at once. No one denies that is an infective disease, and that the infection is by a special organism, though I rather suspect that in some of the most troublesome cases we have to deal not with a pure culture, but with a mixed infection. Again, we find that patients recovering from scarlet fever will sometimes develop in one or more joints a pretty severe arthritis. And the question arises, Is this due to the special organism or not? Probably it differs from the two preceding micro-organisms, and is therefore a third variety of undeniable infection not due to the special germ. Though the infection of scarlet fever is not the direct cause of the arthritis, it is an indirect cause in having produced in the tonsils a condition favourable to the absorption of micro-organisms which may be seeking an entrance to the body. This channel of infection is one which is frequently navigated by micro-organisms, and it behoves us to be careful how we open it up by swabbing the throat, as we may be facilitating the absorption of micro-organisms.

Now I cannot help thinking that with these three forms of infection staring us in the face we must be led to the conclusion that in all probability all the other forms of acute and subacute, if not chronic, joint inflammations are, to say the least of it, the result of infection.

It is interesting to note how alike in the periods and signs are the joints in the early stages of the attack of these different organisms. What we see of the disease is not so much the attacking party as the effect of the resistance. In a great many diseases we mistake the efforts of the body to resist infection for the disease itself, and in many cases we assist the attacking force by helping to pull down the defensive ramparts thrown up by the body.

So far you will notice I have not used the word gout, nor has it a separate place in my classification; this is because I do not think that it belongs to the first list. It falls as a subdivision of toxic arthritis. Whether it is due to the sudden invasion of the joint by a micro-organism or a toxin, or whether it is due to an infection or toxæmia of the nerve centre which governs the joint, no one can at present say. The origin of the micro-organism is almost certainly in the intestine, and there must be some hereditary or acquired weakness of the nerve centre governing the joints, or of the joint tissues themselves, for there is so marked an indication of there being hereditary predisposition, and we find families in which nearly all, if not all, the members are victims of the disease. Osteo-arthritis is common in the female members of a gouty family. Uric acid is, as you know, considered to play an important part in the arthritis, but is it not possible, and even probable, that the real part played by uric acid is in reducing the alkalinity of the blood and so rendering it less potent to deal with pathogenic micro-organisms? And these micro-organisms must find in the uric acid material which is suitable to their *rôle*, which seems to be to combine the uric acid with soda into biurate of soda, a bio-chemical process.

We find that cases of arthritis are by no means uncommon after confinements, and after curetting operations it is not uncommon to find arthritis of varying severity. Perhaps surgeons do not allow a long enough period of time to elapse before they pronounce their patients to be well and allow them to cease, or at any rate be lax in, their employment of antiseptic precautions. The endometritis for which the curetting was performed may have been the result of infection either by a pure culture or a mixture of pathogenic germs which were on their way to the joints. A degenerating fibroid is a source of infection for which we ought always to be on the look-out, and I could wish that gynæcological surgeons who advise that fibroids should be left alone and not operated on would take more into consideration the risks that women run from the degeneration of these tumours at a time when no operation could very well be undertaken.

The nose offers a ready method of entrance for germs, and of course they are in abundance in the air which is passing over the mucous membrane, and a diseased membrane is a ready breeding-ground for them. The pharynx, and particularly the tonsils, should be carefully examined and a history obtained as to whether there has been any antecedent sore throat. An otitis media may be a cause, and is one which is apt to be overlooked if the acute stage occurred some considerable time before. I saw one case in which I am pretty certain that a very severe attack of acute infective arthritis was due to the lady's personal attendant having an old-standing otorrhœa. Some suppurating wound may be the cause, and recently I saw a case which had followed an operation for hernia, in which a portion of the wound had become infected. The bladder is a source of infection, and I believe that in morbus coxis senilis it is frequently the source of infection, and if we can get the condition cured in the early stage we have some chance of curing one of the most intractable of diseases. Before I leave the urinary system let me draw your attention to the possibility of a balanitis being a source of infection.

And now I come to what I consider a most important source of infection, because it is one which very commonly produces other infective ailments, and because it is one which, though generally recognised, is not sufficiently investigated, if I may judge by the few cases in which I have found that it has been fully inquired into among the many cases which have come under my notice. I refer to the infection derived from the mouth.

I am not at all sure that I did not select the subject for a lecture because the day on which I received the invitation to deliver one I overheard a tall, strong gentleman make the following remark outside the Royal Baths: "I have had a regular day of it—an hour with a masseur, an hour with the dentist, and now I am going to have a bath." I thought to myself, "My friend, if you had had your hour with the dentist six months ago, probably you would not now require either the masseur or the bath." And the idea presented itself to me that arthritis of septic origin would perhaps not be an uninteresting subject for my lecture. I have seen many excellent results from attention to and extraction of teeth which have been the cause of infection. A young lady came under my care eighteen months ago who was a good example of what is called rheumatoid arthritis, but which I prefer to call sub-acute infective arthritis. She could not get out of bed without assistance and could not walk without help. Her hands were so crippled she could do but little to assist in dressing herself. She had come from Scotland, and so I felt bound to give her some of the ordinary bath treatment. I felt certain, however, that until she had her teeth extracted she would receive but little benefit. She, however, improved somewhat, which enabled her the better to undergo the operation of having all her teeth out when she got home. She returned this summer able to walk very well and with quite useful hands. During the last season I saw a lady who was suffering from what was called rheumatism affecting the muscles of the back; it struck me as a case of dental affection, but she resented any imputation on her teeth.

She sent for me one day as her face was swollen and she had a gumboil. I sent her to a dentist, whose treatment effected a cure which baths and waters could not. I believe that in the cases of people who wear artificial teeth the mouth is often a cause of infection, and I am in the habit of ordering the teeth to be soaked all night in a weak solution of formalin and the mouth to be washed night and morning with some weak antiseptic.

Suppuration at the sides of the nails may be a cause which escapes our notice—for instance, the suppuration attending on an ingrowing toe-nail. A suppurating corn, and particularly a soft corn, between the toes should be looked for, and indeed if the toilet of the feet has been neglected the importance of attending to it should be insisted upon, and the soap and water be reinforced by some antiseptic so that lost time may be recovered as speedily as possible. Septic foci in the skin from wounds or infection should be sought for.

I am afraid that you will think that this is a terribly long and varied inquiry to have to make, but a good deal of it can be guided by your observations. Remember to make your inquiry before examining your patient, otherwise the patient is apt to think that you have not been able to make up your mind as to what was the matter with him.

### Treatment.

I think that of all forms of local treatment the Bier hyperæmic treatment, either by elastic bandage or vacuum chambers, is perhaps the best. Of course the treatment is rather troublesome and expensive, as it requires the constant attendance of a well-trained nurse, at any rate during the first week of treatment. Intelligent patients may, however, get very expert in applying the treatment to themselves when they understand the rationale of the method. Next, I should place the hot-air treatment by one of the several methods which are employed. This treatment is more comforting and has the advantage of being much more easily applied; and here again the patient can often employ either a home-made or a hired apparatus himself with or without the services of a nurse. If there is any possibility of there being an infection of the joint by a virulent pyogenic organism, the greatest care will be required. I have never attempted to treat these. Dr. Meyer says regarding them that "a strict and refined diagnosis is the first postulate; the ability to carry out any major operation that may become necessary, the second." I think that that speaks volumes.

I have employed the treatment a good deal in cases which were not likely to call upon my capacity for performing major operations, and I have certainly found the treatment beneficial. I think that even the very chronic arthritides which produce Heberden's nodes are benefited by wearing india-rubber rings of the right size.

The condition of the vagina and uterus should receive attention, and douching with hot normal saline will be found very useful.

Physiologists long ago demonstrated to us the marked physiological difference which exists between plain water and water which contains .6 per cent. of chloride of sodium. They have shown us that if a piece of living tissue be placed in plain water it is rapidly acted upon and its integrity destroyed. Whereas if placed in the normal saline it may be examined under the microscope as living tissue. It is remarkable how long it was before the importance of this was recognised, apart from the original application of the discovery to microscopical work. Its very simplicity probably obscured the value of the observation. It was long before any practical use for this simply constructed fluid was found, but now it is well known it is largely employed for intravenous and interstitial injections, because it is not inimical to living tissues, but almost indeed normal to them. The great difference between ordinary hot-water baths and those from medical springs has long been known. Their efficacy has generally been attributed to other ingredients which probably have little to do with it. This I believe to be a partial and simple explanation of the good produced by mineral-water bathing and of the value of sea bathing, where, in addition, the skin is stimulated by an excess of salt.

